# A genomic exploration identifies mechanisms that may explain adverse cardiovascular effects of COX-2 inhibitors

**DOI:** 10.1038/s41598-017-10928-4

**Published:** 2017-08-31

**Authors:** Ingrid Brænne, Christina Willenborg, Vinicius Tragante, Thorsten Kessler, Lingyao Zeng, Benedikt Reiz, Mariana Kleinecke, Simon von Ameln, Cristen J. Willer, Markku Laakso, Philipp S. Wild, Tanja Zeller, Lars Wallentin, Paul W. Franks, Veikko Salomaa, Abbas Dehghan, Thomas Meitinger, Nilesh J. Samani, Folkert W. Asselbergs, Jeanette Erdmann, Heribert Schunkert

**Affiliations:** 1 Institute for Cardiogenetics, University of Lübeck, 23562 Lübeck, Germany; 2 DZHK (German Research Center for Cardiovascular Research), partner site Hamburg/Lübeck/Kiel, 23562 Lübeck, Germany; 3 University Heart Center Lübeck, 23562 Lübeck, Germany; 4 Department of Cardiology, Division Heart and Lungs, University Medical Center Utrecht, 3584 Utrecht, The Netherlands; 5 Deutsches Herzzentrum München, Technische Universität München, 80636 München, Germany; 6 University of Michigan, Dept of Biostatistics, 1415 Washington Hts, Ann Arbor, MI 48104 USA; 7 Institute of Clinical Medicine, Internal Medicine, University of Eastern Finland and Kuopio University Hospital, 70210 Kuopio, Finland; 8 Preventive Cardiology and Preventive Medicine, Center for Cardiology, University Medical Center Mainz, Mainz, Germany; 9 Center for Thrombosis and Hemostasis, University Medical Center Mainz, Mainz, Germany; 10 DZHK (German Center for Cardiovascular Research), Partner Site RhineMain, Mainz, Germany; 11 Department of General and Interventional Cardiology, University Heart Center Hamburg-Eppendorf, Hamburg, Germany; 12 Uppsala Clinical Research Center, Uppsala Science Park, MTC, SE-752 37 Uppsala, Sweden; 13 Genetic and Molecular Epidemiology Unit, Department of Clinical Sciences, Lund University, Skåne University Hospital Malmö, Malmö, Sweden; 14 THL-National Institute for Health and Welfare, POB 30, Mannerheimintie 166, FI-00271 Helsinki, Finland; 15 Department of Epidemiology, Erasmus University Medical Center, 3000 CA Rotterdam, The Netherlands; 16 Institute of Human Genetics, Helmholtz Zentrum München, German Research Center for Environmental Health, 85764 Neuherberg, Germany; 17 DZHK (German Center for Cardiovascular Research), partner site Munich Heart Alliance, 80636 Munich, Germany; 18 Institute of Human Genetics, Technische Universität München, 81675 Munich, Germany; 19 Deparment of Cardiovascular Sciences University of Leicester and NIHR Leicester Cardiovascular Biomedical Research Unit, Glenfield Hospital, Leicester, LE3 9QP UK; 20 Institute of Cardiovascular Science, faculty of Population Health Sciences, University College London, London, United Kingdom

## Abstract

Cyclooxygenase-2 inhibitors (coxibs) are characterized by multiple molecular off-target effects and increased coronary artery disease (CAD) risk. Here, we systematically explored common variants of genes representing molecular targets of coxibs for association with CAD. Given a broad spectrum of pleiotropic effects of coxibs, our intention was to narrow potential mechanisms affecting CAD risk as we hypothesized that the affected genes may also display genomic signals of coronary disease risk. A Drug Gene Interaction Database search identified 47 gene products to be affected by coxibs. We traced association signals in 200-kb regions surrounding these genes in 84,813 CAD cases and 202,543 controls. Based on a threshold of 1 × 10^−5^ (Bonferroni correction for 3131 haplotype blocks), four gene loci yielded significant associations. The lead SNPs were rs7270354 (MMP9), rs4888383 (BCAR1), rs6905288 (VEGFA1), and rs556321 (CACNA1E). By additional genotyping, rs7270354 at MMP9 and rs4888383 at BCAR1 also reached the established GWAS threshold for genome-wide significance. The findings demonstrate overlap of genes affected by coxibs and those mediating CAD risk and points to further mechanisms, which are potentially responsible for coxib-associated CAD risk. The novel approach furthermore suggests that genetic studies may be useful to explore the clinical relevance of off-target drug effects.

## Introduction

Selective COX-2 inhibitors (coxibs) display non-steroidal anti-inflammatory effects, which are widely used to treat chronic pain syndromes. However, long-term administration of coxibs has been found consistently to increase risk of cardiovascular events including myocardial infarction and coronary death^1^. Given such severe adverse effects, their use is controversially discussed in the United States and Europe^2^. In fact, several coxibs, such as rofecoxib, have been withdrawn from the marked for that reason^3^.

The recently published PRECISION trial evaluated the cardiovascular safety of celecoxib and reported no increased risk as compared to two nonselective NSAID^4^. However, the results have been controversially discussed for a number of reasons^5, 6^. For example, almost 70% stopped taking the medication during the two years of follow-up, which was entirely lost in about 25% patients^4^. Moreover, celecoxib was somewhat less effective than its comparators; most likely due to the relatively low dose of celecoxib used in this trial (about 200 mg/day). By contrast, studies that have shown an increase of coronary risk used higher doses (400–800 mg). Perhaps most importantly, the variants identified in our study are located in genes that are downstream of COX2 and thus affected in a similar way by the three drugs tested. Hence, our data may be relevant for NSAID in general and add to the long standing discussion of the underlying mechanisms related to both intended (analgesic) and unintended (cardiovascular) effects.

Their principle mechanism of action is to selectively inhibit the cyclo-oxygenase 2 isoform (COX-2) to reduce prostaglandin I2 and prostacyclin pro-inflammatory effects. In contrast to non-selective Cox-inhibitors, coxibs do not lower COX-1 derived thromboxane production. Since thromboxane activates platelets, selective Cox-2 inhibitors may affect unfavorably the prostacyclin (antithrombotic)/thromboxane (prothrombotic) ratio, which may explain their thrombo- and atherogenicity, and their blood pressure increasing effects^3^. However, coxibs display numerous other (pleiotropic or off-target) effects, which likewise could add to the untoward safety profile of the drugs^3^. For example, coxibs may suppress NO production, which has been related to CAD risk by genetic means^7^. Moreover, other Cox-inhibitors not affecting the prostacyclin/thromboxane ratio may also increase coronary event rates^2^. Thus, the precise mechanisms explaining cardiovascular risks of coxibs are not proven definitively^2^.

Genetic variants affecting disease risk can facilitate identification of drug targets^8, 9^. Likewise, variants may point to potential adverse effects, if risk alleles and drugs have similar functional implications^10, 11^. Here, we reversed this approach and systematically explored known molecular targets of coxibs for signals in genome-wide association studies (GWAS) on CAD.

The starting point of our analysis was to identify genes or gene products reported in the *Drug Gene Interaction Database* ^12^ to interact with coxibs. Considering all genes interacting with coxibs we aimed to filter out those with the potential to affect the CAD risk related to these drugs specifically by searching respective genes for genomic signals indicating CAD risk. The underlying idea is that single nucleotide polymorphisms (SNPs) may mimic drug effects in disturbing the function or regulation of a gene product, and therefore – like the drug – associate with CAD risk, even if the effect sizes might vary. The enormous statistical power of contemporary genomic analyses was thereby used to scrutinize the spectrum of pleiotropic coxib effects for those that may contribute to the increased CAD risk of long-term users of these frequently prescribed drugs. Remarkably, the strategy also led to successful identification of novel variants genome-wide significantly associated with CAD risk.

## Methods

**Figure 1:**
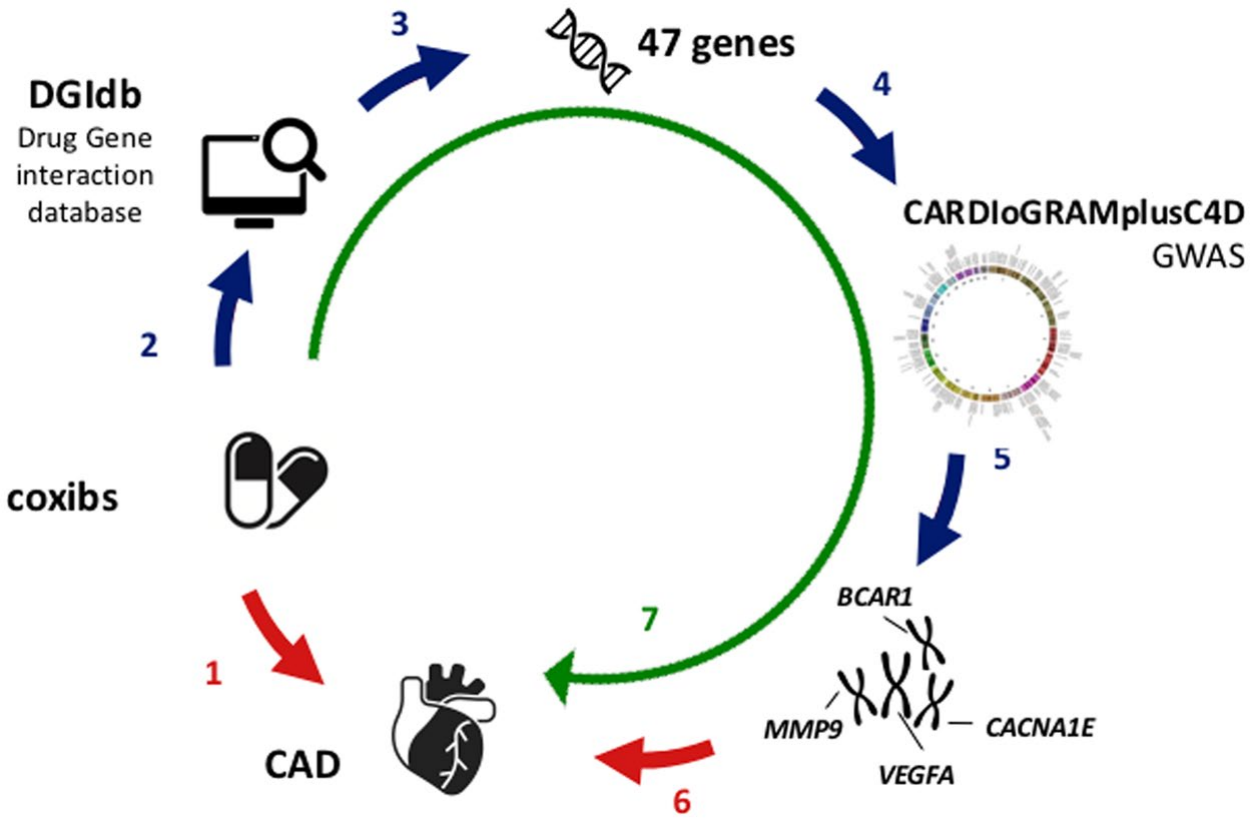
Experimental Strategy: 1. Cox 2 inhibitors (coxibs) are known to increase coronary risk. 2. All genes known to be targets of coxibs were extracted from the Drug-Gene Interaction Database (DGIdb). 3. DGIdb revealed 47 genes that interact with coxibs. 4. All common variants at the chromosomal regions representing the 47 genes were subjected to a large-scale association study. 5. Four genes displayed significance for association with CAD risk. 6. These genes are candidate risk genes for CAD. 7. It may be hypothesized that the genes affected by coxibs and here shown to associate with CAD risk participate in the adverse effects of the drugs. Some drawings were obtained and used under license from Shutterstock.com (https://www.iconfinder.com/licenses/basic). Images can be found under: https://www.shutterstock.com/image-vector/chromosomes-vector-icon-style-flat-symbol-323629910?src=library, https://www.shutterstock.com/image-vector/organ-heart-icon-310580756, https://www.shutterstock.com/image-vector/dna-icon-397249525, https://www.shutterstock.com/image-vector/pills-medication-vector-icons-set-131402543?src=library, https://www.iconfinder.com/icons/240302/find_computer_find_desktop_look_for_desktop_search_search_desktop_icon#size=128.

The principle approach taken in this study is illustrated in Fig. 1. The first step of the pipeline was to identify genes or gene products reported to interact with coxibs. For this we downloaded the Drug Gene Interaction Database version 2.22 (DGIdb)^12^. This database aims to capture genes that are known to be targeted by drugs. It is rather inclusive in that liberal criteria are used to allow entry in this database^12^. Thus, it may also include gene or gene products with relatively weak evidence for drug interaction. Despite this noise, we decided not to perform any manipulation on the database to avoid a bias introduced by *a priori* hypothesis. With this strategy, we expect that at least some of the genes reported in the database reflect true interactions which are interesting for genetic interrogation of coxib-related cardiovascular side effects.

We included all genes reported to interact with celecoxib, etoricoxib, lumiracoxib, parecoxib, rofecoxib, and valdecoxib. The genes reported in DGIdb are shown in supplementary Table 2. This table also shows the complete output of the DGIdb query. We identified all SNPs within 200-kb surrounding the genes whose products are affected by these drugs. The 200-kb window was selected to capture most genetic variants with potential regulatory effects.

### GWAS datasets

Having identified the drug gene interactions, we subsequently screened respective genes for association signals in the CARDIoGRAMplusC4D 1000 G GWAS meta-analysis on CAD^13^ and additional non-overlapping datasets from CARDIoGRAM^14^ and German MI Family Studies V (GerMIFS V). The CARDIoGRAMplusC4D 1000Genomes meta-analysis data set consists of 47 GWAS studies including data from 60,801 cases and 123,504 controls. The GWAS are imputed with the 1000Genomes phase I integrated haplotypes from December 2012 (ftp://ftp.1000genomes.ebi.ac.uk/vol1/ftp/release/20110521/) (for Methods see Nikpay *et al*.^13^).

We combined this data set^13^ with GWAS from CHARGE^15^, deCODE CAD^16^, CADomics^14^, DILGOM^17^, EPIC^7^, FRISC II GLACIER^18^, METISM^19^, MORGAM FIN^20^, MORGAM FRA^20^, MORGAM GER^20^, MORGAM ITA^20^, MORGAM UNK^20^, PMB^7^, PopGen^21^, SCARF SHEEP^7^, and STR^22^ that have been previously reported in references Schunkert *et al*.^14^ and/or Deloukas *et al*.^7^. Moreover, we included data from GWAS not reported before, i.e. GerMIFS V. See detailed information in supplementary Table 1. In total, this combined dataset consists of 84,813 cases and 202,543 controls.

The SNPs identified in the first stage of analysis were genotyped in additional 2,496 cases and 1,505 controls from the GerMIFS VI study (unpublished).

### Statistical Methods

In the exploratory analysis of SNPs, we assumed significance for association with CAD after Bonferroni correction for the number of independent SNPs tested. We calculated the number of independent SNPs based on the linkage disequilibrium (LD) between SNPs. This method is analogue to the one proposed by Nyholt^23^. Nyholt *et al*. proposed a method to correct for multiple testing based on pairwise LD between SNPs. Because Nyholts approach, which uses spectral decomposition of pairwise LD matrices, is computationally demanding, we correct using the number of haplotype blocks. The number of independent SNPs was estimated based on the number of haplotype blocks calculated with PLINK software using the default setting of the blocks command. We calculated the haplotype blocks using the GerMIFS IV study. We do not have individual genotype data from all studies but expect this GWAS to be representative. For a total of 81,703 SNPs within the 47 gene regions tested, we identified 3,131 haplotype blocks and defined significance according to Bonferroni at a p-value threshold of 1 × 10–5.

### Meta-analysis

Logistic regression, assuming an additive model, was performed on all single study data. We performed logistic regression with all genotype data available. Individuals with no genotype data or with poor quality genotype data were excluded from regression analysis. All analyses were adjusted for sex and age. Age was defined as the recruitment age for controls and the event age for cases. For meta-analysis, we calculated an ‘inverse variance weighting’- fixed-effects and a random effects model^24^, depending on the heterogeneity between the studies. For heterogeneity calculation Cochran’s Q was used. Threshold for heterogeneity was phet <0.01. We combined the CARDIoGRAMplusC4D 1000Genomes meta-analysis data set and non-overlapping CARDIoGRAM, GerMIFS V, and GerMIFS VI studies using an ‘inverse variance weighting’-fixed-effects model and combined effects and p-values were reported. In total we evaluated the genomic data from 87,309 CAD patients and 204,048 controls.

### Functional annotation of SNPs and genes

To evaluate the functional implication of the SNPs, we identified all SNPs with an LD of r^2^ > 0.8 to the locus lead SNP using the HaploReg version 3 database^25^. We then used the Cardiogenics data set^26^ for a systematic search for expression quantitative trait loci (eQTLs) affecting monocyte and macrophage expression, the publicly available data from Westra *et al*.^27^, GTeX^28^ and over 100 studies included in the Genome-Wide Repository of Associations between SNPs and Phenotypes (GRASP) database^29^. In addition, we used HaploReg^25^ to locate SNPs in promoter and enhancer regions and performed a literature search for gene functions using Pubmed.

### Identification of pleiotropic cardiovascular effects of SNPs

We used the GRASP database to screen for association signals for CAD-related phenotypes at the four loci displaying association with CAD. The GRASP database contains the association results of around 1,390 GWAS^29^ and is the most comprehensive GWAS database. We searched for reported associations with CAD-related traits in the GRASP database for all SNPs with LD of r^2^ > 0.8 with the lead SNPs. We used p* < *1 × 10^−3^ for CAD-related traits to avoid false positive associations.

### Test for enrichment

To test for enrichment, we downloaded the GSEA curated pathways database (MSigDB version c2.cp.v5.1)^30^ which comprises a total of 1330 pathways. We compared the number of eSNPs displaying a significant effect – after the Bonferroni correction p < 1 × 10^−5^ as used in our main analysis – for CAD risk in these pathways with respective eSNPs within the coxib gene subset using the Fisher exact test. SNPs were annotated the using the publicly available eQTL databases listed above.

## Results

**Figure 2:**
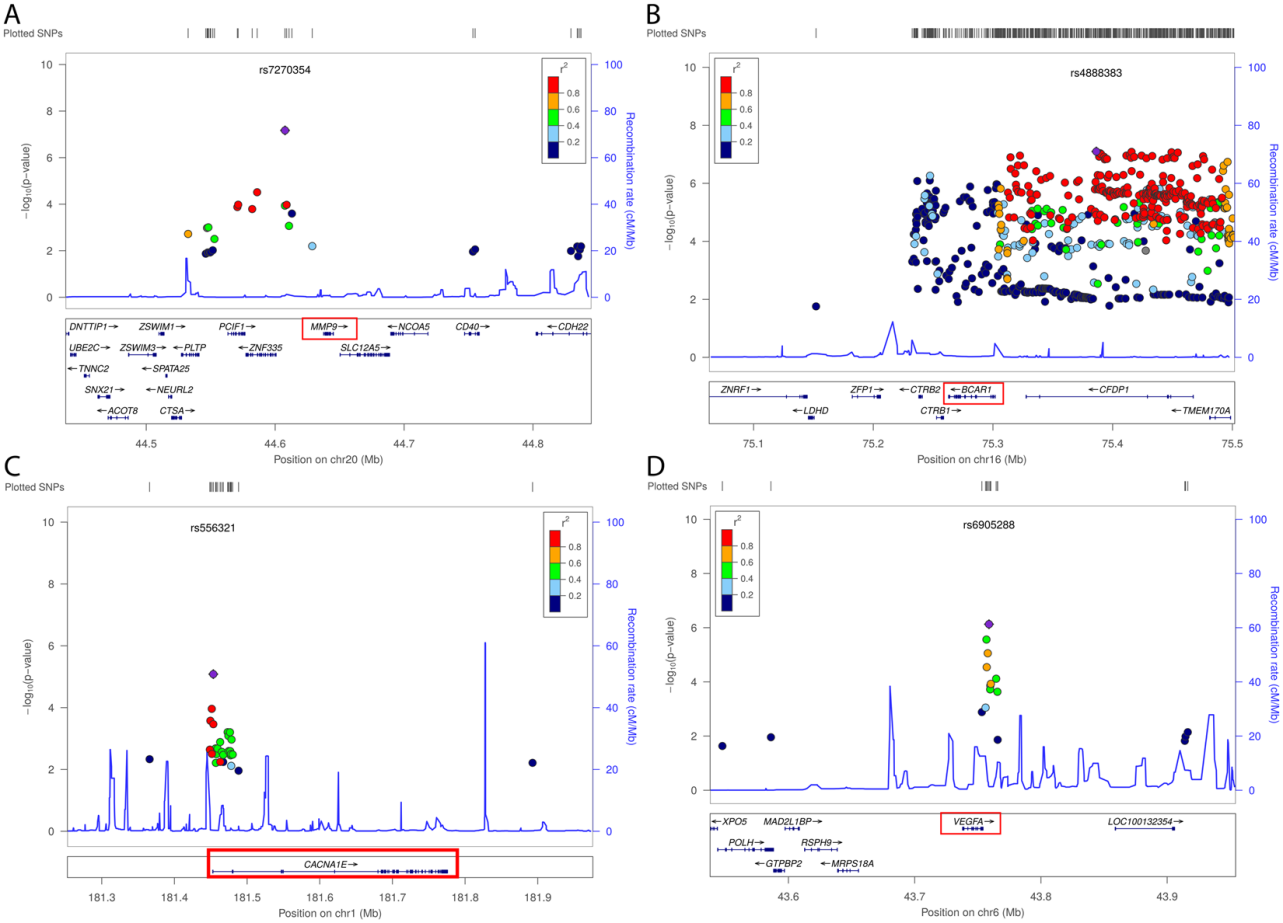
Association signals for at the chromosomal regions of coxib genes (**A**) *MMP9*, (**B**) *BCAR1*, (**C**) *CACNA1E*, and (**D**) *VEGFA* using Locus Zoom^53^.

We identified in the publicly available *Drug Gene Interaction Database* 47 genes or gene products to interact with coxibs^12^ (supplementary Table 2). We studied the 200-kb region surrounding respective genes in a combined 1000Genomes imputed meta-analysis (data from CARDIoGRAM and CARDIoGRAMplusC4D) to search for SNPs showing association with CAD^13^. Correcting for multiple testing accounting for haplotype blocks, we considered SNPs significant with a p-value lower than 1 × 10^−5^. In this way, we revealed a total of four loci associated with CAD (Table 1 and Fig. 2 and supplement Table 3).

**Table 1:** Association signals for CAD within 200 kb of genes reported for coxib/gene product interactions.

Gene	Lead SNP	CAD risk allele	Frequency risk allele	OR risk allele	P-value	P-value combined analysis	Relationship of coxib to gene/gene product*	Regulatory effect (Histone marks)
*BCAR1*	rs4888383	T	0.57	1.05	7.99*10^−08^	2.98*10^−08^	inhibition	Promoter/Enhancer
*MMP9*	rs7270354	A	0.15	1.06	6.75*10^−08^	3.34*10^−08^	decreased expression	Promoter/Enhancer
*VEGFA*	rs6905288	A	0.60	1.04	7.44*10^−07^	8.86*10^−07^	decreased expression	Enhancer
*CACNA1E*	rs556321	C	0.16	1.05	8.26*10^−06^	8.85*10^−06^	inhibition	Promoter/Enhancer

For each gene, the lead SNP is shown. The regulatory effects were annotated using Haploreg^25^.

In the coxib pathway we identified 43 genes out of which 3 (or 7%) were found to have eSNPs displaying a significant effect – after Bonferroni correction p < 1 × 10^−5^ – for CAD risk. For comparison, in GSEA curated pathways out of 7076 genes only 1.5% of genes had respective eSNPs with a comparable signal for CAD risk. Hence, the set of genes on which our approach was focused was significantly enriched – by Fisher exact p-value of 0.03 – for genes with an eSNPs showing a signal for CAD.

The genes affected by coxibs and located within the loci showing SNP association for CAD are the vascular endothelial growth factor A (*VEGFA*), matrix metalloproteinase-9 (*MMP9*), breast cancer anti-estrogen resistance protein 1 (*BCAR1*) and the calcium channel, voltage-dependent, R type, alpha 1E subunit (*CACNA1E*). For the lead SNPs tagging these genes, we performed additional genotyping of 2,496 cases and 1,505 controls.

The A allele of the *VEGFA* lead SNP, rs6905288, is associated with CAD with a p-value of p = 8.86 × 10^−7^ (OR 1.04) in our data. In the GRASP database, this SNP shows genome-wide significant association with waist hip ratio and suggestive association with HDL cholesterol level, triglycerides, and systolic and diastolic blood pressure, respectively (for references and further information please see the supplement Table 4). The lead SNP is found in enhancer regions, and is associated with a decreased expression of VEGFA in adipose tissue^31^.

The A allele of the *MMP9* lead SNP, rs7270354, is located upstream of the gene and is genome-wide significantly associated with CAD (p = 3.34 × 10^−8^, OR 1.06). The lead SNP and linkage disequilibrium (LD) SNPs are found in enhancer regions in several cell types. However, neither this SNP nor a SNP in LD (r^2^ > 0.8) is associated with another CAD related phenotype in GRASP. The CAD risk allele is associated with increased expression of MMP9 in whole blood^27^. The effect of the CAD risk allele is opposite of the effect of coxibs on the gene. However, it is well established that SNPs showing eQTL effects, can have opposite effect in different tissues^32^. Hence, the identified eQTL effect demonstrates the regulatory effect of the SNP on MMP9. Further studies are needed to identify the effect of this SNP in CAD relevant tissue as for instance smooth muscle cells.

Lead SNP, rs4888383, is located upstream of the gene *BCAR1* and shows genome-wide significant association with CAD (p = 2.98 × 10^−8^, OR T 1.05). SNP rs4888383 is located in a potentially functional promoter region, with the CAD risk allele (T), and SNPs in high LD (r^2^ > 0.8), being linked to reduced expression of *BCAR1* in the esophagus mucosa (GTeX). The LD SNP rs4888378, was previously associated with carotid intima media thickness (p = 6.5 × 10^−7^) and CAD (p = 6.53 × 10^−6^). In addition, the lead SNP shows suggestive association with systolic and pulse pressure, urinary albumin to creatinine ratio, and microalbuminuria (for references and further information please see supplementary Table 4).

The C allele of the *CACNA1E* lead SNP, rs556321, and several LD SNPs span the promoter of the gene. It is associated with CAD with a p-value of p* = *8.85 × 10^−6^ (OR 1.05). Genetic variants in *CACNA1E* are reported to play a role in hypertension. LD SNPs of rs556321 are associated with blood metabolite concentrations and obesity. The CAD risk allele is associated with decreased expression of *CACNA1E* in blood (for references and further information see supplementary Table 4).

**Figure 3:**
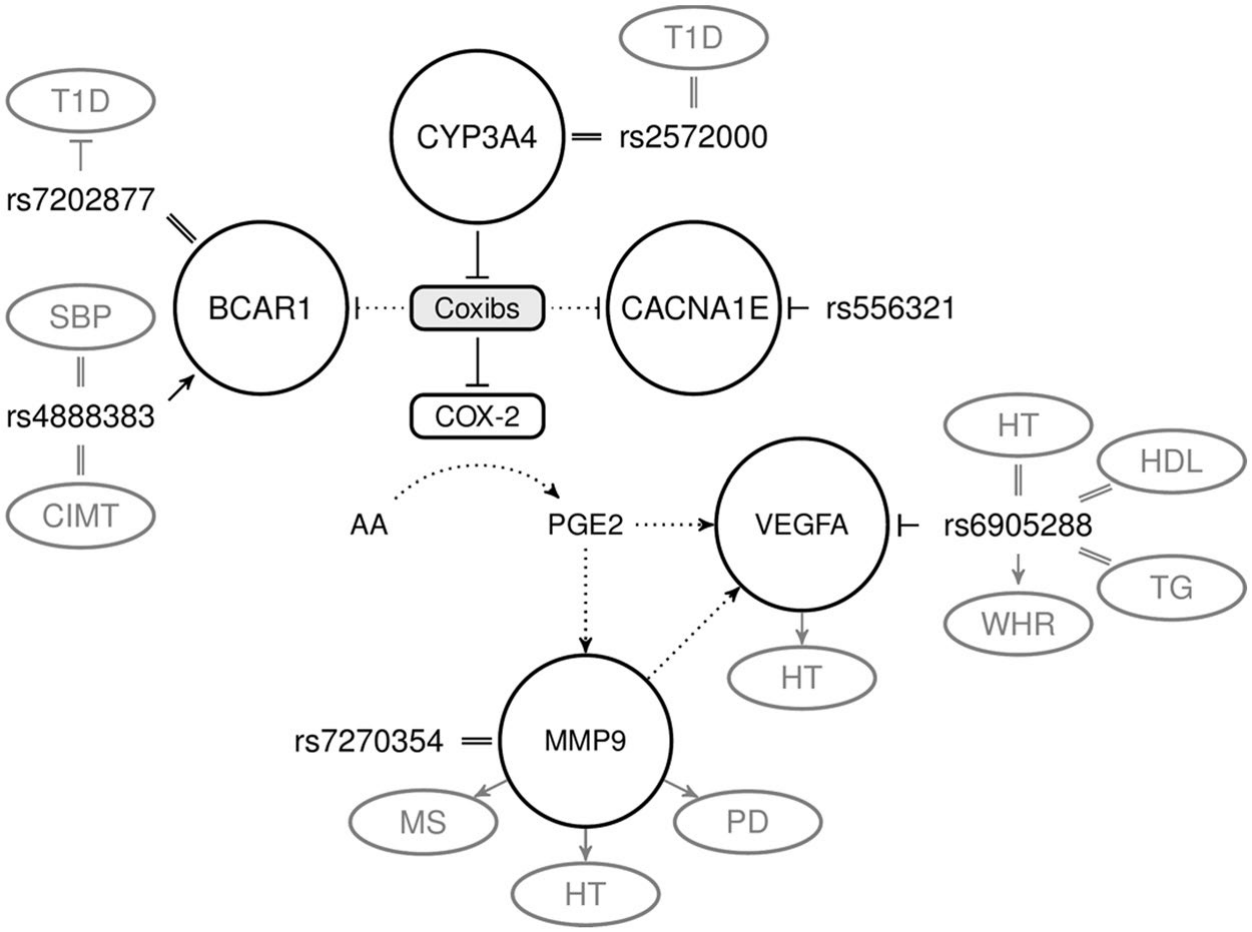
Pharmacodynamic effects of coxib treatment related to the genetic association signals. All SNPs and their functional implication are shown based on the CAD risk allele. The rs numbers indicate the lead SNPs and their hypothesized function. rs6905288 is strongly associated with waist-to-hip-ratio and rs7202877 with type 1 diabetes (T1D). Suggestive associations were reported for rs4888383 with carotid intima media thickness (CIMT) and systolic blood pressure (SBP), for rs6905288 with hypertension (HTN), HDL-cholesterol, and triglycerides (TG), and for rs556321 with obesity (OB) and blood metabolites (BM). CAD risk allele rs556321 is associated with decreased expression of *CACNA1E*, rs4888383 is associated with decreased expression of BCAR1 and rs6905288 is associated with decreased expression of VEGFA. Functional links: ↑ (induction), ┬ (inhibition), ║(unknown effect). Dotted lines indicate potential intermediate functional link. AA: arachidonic acid, COX-2: Cyclooxigenase-2, PGE2: prostaglandin E2, VEGFA: vascular endothelial growth factor A, MMP9: matrix metalloproteinase-9, BCAR1: breast cancer anti-estrogen resistance protein 1, CACNA1E: calcium channel, voltage-dependent, R type, alpha 1E subunit. PD: Plaque disruption, MS = Metabolic Syndrome, T1D: type 1 diabetes, CIMT: carotid intima media thickness, SBP: systolic blood pressure, HTN: hypertension, HDL: HDL-cholesterol, TG: triglycerides, OB: obesity, BM: blood metabolites. For references see supplement.

Figure 3 integrates the four genes in the functional pathways affected by coxib administration. It illustrates that VEGFA and MMP9 are downstream of the principle mechanism of COX-2 inhibition and thus may represent a class effect. BCAR1 and CACNA1E are not related to the intended pharmacological action and may thus represent off-target effects.

## Discussion

A data-base on drug-gene interactions lists 47 genes or gene products to be affected by administration of coxibs^12^, a class of drugs known for prominent coronary side effects. Analysis of the chromosomal loci harboring respective genes identified two novel loci with genome-wide significant association and two loci with robust association signals for CAD. In conjunction, it appears that coxibs affect several gene products that also play a role in modulating genetic risk of CAD. With respect to the pharmacological profile of coxibs, these data point to potential novel mechanisms for the increased myocardial infarction risk observed in long-term users of this medication.

The reason for the multitude of genes affected by inhibition of COX-2 relates in part to the variety of downstream signaling events caused by reduction of prostaglandin synthesis. *VEGFA* and *MMP9* may be involved via this primary drug effect. *BCAR1* may be affected by coxibs via other, pleiotropic mechanisms, but – like *VEGFA* and *MMP9* – this gene has been studied functionally in the context of atherosclerosis before^33–36^. Thus, detection of robust genetic signals for association with CAD within these genes appears to be plausible.

Celecoxib is reported to suppress the expression of *VEGFA* ^37^. VEGFA is an important signaling molecule that mediates vasculogenesis, angiogenesis, and vascular maintenance^38^. VEGFA furthermore augments NO levels and, hence, may ameliorate platelet aggregation and vasoconstriction^39^. Inhibition of VEGFA is reported to lead to hypertension^35^. The lead SNP at the VEGFA locus showed multiple associations with coronary risk factors further corroborating its role in CAD.

Celecoxib is reported to inhibit the production of MMP9 in several cell types^40^. MMP9 is a zinc-dependent endopeptidase with tissue-specific expression known to up-regulate VEGFA^34, 41^. On the other hand, higher levels of MMP9 have been reported in patients with CAD^33^ and metabolic syndrome^42^. In addition, MMP9 has been linked with hypertension^43^, arterial stiffness^43^ and is reported to induce acute plaque disruption^44^. Further, MMP activation has been related to decreased aortic elasticity in mice via prostaglandin E2 receptor signaling^45^.

Celecoxib is reported to inhibit BCAR1^46^ which belongs to the CAS family of scaffolding proteins substrate and is often linked to several forms of cancer^47^. In addition, the gene is reported to be associated with focal adhesions, regulation of smooth muscle cell migration, carotid intima media thickness and CAD^48^. It appears to be activated downstream through the angiotensin II pathway^49^. Interestingly, *BCAR1* overexpression has been associated with celecoxib resistance in colorectal cancer^46^.

Celecoxib is reported to inhibit L-type calcium channels in a COX-2 independent pathway^50^. CACNA1E mediates the cell surface membrane potential and affects several cellular processes such as contraction and gene expression^51^. The lead SNP at the CACNA1E locus showed associations with other coronary risk factors. CACNA1E inhibition is also reported to lead to hypertension^36^ and hence, as VEGFA, is a strong candidate to explain the CAD risk of coxibs. The CAD risk SNP representing the CACAN1E locus is associated with reduced expression of the gene, which matches with the direction of effect reported for coxibs.

Taken together, our data indicate mechanistic parallelisms between chronic intake of coxibs and genetic variants affecting CAD risk, both being diverse. In line with our principle observations, we also find a large overlap of coxib related side effects and the traits related to the SNPs (see supplement Table 5). The four genes, identified in this study to associate genetically with CAD risk, are all reported to interact with celecoxib in the DGIdb. The other coxibs, however, might also alter these genes, without being studied in detail so far. Especially *MMP9* and *VEGFA*, downstream of COX-2, are likely to be affected by the other coxibs.

On the cellular level, coxibs have been implicated to modulate angiogenesis, inflammation, cell cycle arrest, and apoptosis^52^, as well as focal adhesions and vascular tone^50^. The interaction between coxibs and the genes associated with CAD by our investigation may reinforce the implications of some of the adverse mechanisms affected by the drugs. Indeed, given the association findings reported in this study it appears plausible that the increased CAD risk is also mediated by mechanisms other than disturbing the prostacyclin/thromboxane ratio. While *VEGFA* and *MMP9* are both downstream COX-2 inhibition, *BCAR1* and *CACNA1E* are affected presumably through independent pathways.

In this work, we defined the risk alleles as those found more frequently in CAD cases. The definition is not based on functional testing and defining the allele more frequently found in controls as protective would be equally valid. However, exploring publically available data bases we observed genotype specific differences in gene expression which might relate to the mechanistic effects. We assigned the SNPs to *VEGFA*, *MMP9*, *BCAR1*, and *CACNA1E* because these were the genes altered by coxibs and because the SNPs are eQTLs for the respective genes. It is, however, possible that the SNPs have effects on other genes as well. Indeed, we have previously demonstrated that regulatory SNPs influence the expression of multiple genes^32^. Hence, we do not exclude other genes to mediate the effect on CAD for the identified loci.

Our study design has several limitations. All our findings are based on associations rather than functional testing. Here we did not test formally whether the effects of coxibs and risk alleles on respective gene products have identical directionality. This may be particularly evident, as we cannot always derive the directionality of the SNPs on expression levels of the genes that are considered as intermediary phenotypes. However, functional tests reported in the literature, as described above, are consistent with the genetic evidence provided here. Finally, we have no information in the large CARDIoGRAMplusC4D database on the intake of pain relieving medication prior to the manifestation of coronary disease. Since the association is depending on the genotype, drugs or other confounding factors are unlikely to inflate the findings. To have an effect on the association, the administration or the metabolism of the drug has to also depend on the genotype. We cannot completely exclude a pleiotropic effect, but we consider it to be very unlikely. Given the conclusive nature of the association findings, first between coxibs and CAD risk^2^, second between coxibs and respective gene products^12^, and third, between genetic variants at respective loci and CAD risk reported here, the data are highly plausible. Particularly, the strength of association with genome-wide significance for some of the gene variants may be considered to be sufficient even in a hypothesis-free analysis.

Taken together, pleiotropic or off-target effects are responsible for some adverse effects of drugs. Studying all genes known to be affected by coxibs, a class of drugs related to a prominent coronary risk, we have identified genomic mediators of CAD risk. The effects of coxibs on these genes might contribute to the unsatisfactory safety profile of this frequently used medication. Last, by exploring the mechanisms underlying the coxibs adverse effects, we revealed genome-wide significant associations for increased risk CAD at two genomic loci.

## Electronic supplementary material

Supplement Tables
