# Supplementary material for: A genomic exploration identifies mechanisms that may explain adverse cardiovascular effects of COX-2 inhibitors
Source: Sci Rep. 2017 Aug 31;7:10252. doi: 10.1038/s41598-017-10928-4 (PMC5579257; doi:10.1038/s41598-017-10928-4)
Supplement: Supplementary file 1 — Supplement Tables [file 41598_2017_10928_MOESM1_ESM.doc]

**A genomic** **exploration identifies mechanisms that may explain adverse cardiovascular effects of COX-2 inhibitors**

Ingrid Brænne^1,2,3^, Christina Willenborg^1^, Vinicius Tragante^4^, Thorsten Kessler^5^, Lingyao Zeng^5^, Benedikt Reiz^1,2,3^, Mariana Kleinecke^1,2,3^, Simon von Ameln^5^, Cristen J. Willer^6^, Markku Laakso^7^, Philipp S. Wild^8,9,10^, Tanja Zeller^2,11^, Lars Wallentin^12^, Paul W. Franks^13^, Veikko Salomaa^14^, Abbas Dehghan^15^, Thomas Meitinger^16,17,18^, Nilesh J Samani^19^, Folkert W. Asselbergs^4,20^, Jeanette Erdmann^1,2,3,*^, Heribert Schunkert^5,17^

^1^Institute for Cardiogenetics, University of Lübeck, 23562 Lübeck, Germany

^2^DZHK (German Research Center for Cardiovascular Research), partner site Hamburg/Lübeck/Kiel, 23562 Lübeck, Germany

^3^University Heart Center Lübeck, 23562 Lübeck, Germany

^4^Department of Cardiology, Division Heart and Lungs, University Medical Center Utrecht, 3584 Utrecht, The Netherlands

^5^Deutsches Herzzentrum München, Technische Universität München, 80636 München, Germany

^6^University of Michigan, Dept of Biostatistics, 1415 Washington Hts, Ann Arbor, MI, 48104

^7^Institute of Clinical Medicine, Internal Medicine, University of Eastern Finland and Kuopio University Hospital, 70210 Kuopio, Finland

^8^Preventive Cardiology and Preventive Medicine, Center for Cardiology, University Medical Center Mainz, Mainz, Germany

^9^Center for Thrombosis and Hemostasis, University Medical Center Mainz, Mainz, Germany

^10^DZHK (German Center for Cardiovascular Research), Partner Site RhineMain, Mainz, Germany

^11^Department of General and Interventional Cardiology, University Heart Center Hamburg-Eppendorf, Hamburg, Germany

^12^Uppsala Clinical Research Center, Uppsala Science Park, MTC, SE-752 37 Uppsala, Sweden

^13^Genetic and Molecular Epidemiology Unit, Department of Clinical Sciences, Lund University, Skåne University Hospital Malmö, Malmö, Sweden

^14^THL-National Institute for Health and Welfare, POB 30, Mannerheimintie 166, FI-00271 Helsinki, Finland

^15^Department of Epidemiology, Erasmus University Medical Center, 3000 CA Rotterdam, The Netherlands.

^16^Institute of Human Genetics, Helmholtz Zentrum München, German Research Center for Environmental Health, 85764 Neuherberg, Germany

^17^DZHK (German Center for Cardiovascular Research), partner site Munich Heart Alliance, 80636 Munich, Germany

^18^Institute of Human Genetics, Technische Universität München, 81675 Munich, Germany

^19^Deparment of Cardiovascular Sciences University of Leicester and NIHR Leicester Cardiovascular Biomedical Research Unit, Glenfield Hospital, Leicester, LE3 9QP, UK

^20^Institute of Cardiovascular Science, faculty of Population Health Sciences, University College London, London, United Kingdom

***Corresponding author:** jeanette.erdmann@uni-luebeck.de

Supplementary Table 1 4

Supplementary Table 2 5

Supplementary Table 3 13

Supplementary Table 4 13

Supplementary Table 5 13

# Supplementary Table 1

| **Study** | **Phenotype** | **Full name** | **Sample Size Cases/controls** | **M(SD) Age** | **Female (%)**  **Cases/controls** | **Ref** | **Genotyping Plattform** | **Ethnicity** | **Demographic information** |
| --- | --- | --- | --- | --- | --- | --- | --- | --- | --- |
| ***CARDIoGRAM*** |  |  |  |  |  | PMID: 21378990 |  |  |  |
| CHARGE | CAD/MI | Cohorts for Heart and Aging Research in Genomic Epidemiology | 2287/22024 | 60.0(7.9)/ 63.1(8.0) | 33.4/ 59.6 | PMID:  20031568 | Illumina HH300/HHCNV370 / Illumina Infinium HumanHap 550K  Affymetrix 6.0  Affymetrix 500K | European | Island/  The Netherands/ United States |
| deCODE CAD | CAD/MI |  | 6640/27611 | 74.8 (11.8)/ 53.7(21.5) | 36.3/ 61.9 | PMID:  17478679 | Illumina HH300/HHCNV370 | European | Iceland |
| CADomics | CAD/MI | Coronary Artery Disease and Omics | 2078/2952 | 60.8(10.1)/ 55.3(10.8) | 21.9/ 50.5 | PMID: 21378990 | Affymetrix Genome-Wide Human SNP Array 6.0 | European | Germany |
| ***CARDIOoGRAMplus C4D MetaboChip*** |  |  |  |  |  | PMID: 23202125 |  |  |  |
| DILGOM | CAD/MI | The Dietary, Lifestyle, and Genetic determinants of Obesity and Metabolic syndrome study | 147/3844 | 56.6(9.5)/ 51.7(13.6) | 54.4 | PMID: 21179014 | Illumina Cardio‐ Metabochip | European | Finland |
| EPIC | CAD/MI | The European Prospective Investigation into Cancer | 1526/2409 | 71.8(8.18)/ 60.3(9.3) | 47.4 | PMID: 23202125 | Illumina Cardio‐ Metabochip | European | England |
| FRISC II - GLACIER | CAD/MI | Fragmin and Fast Revascularization during Instability in Coronary Artery Disease Gene x Lifestyle interactions And Complex traits Involved in Elevated disease Risk | 2937/6310 | 66.2(9.8)/ 50.5(9.3) | 49.8 | PMID: 20870969  PIMD: 10475181 | Illumina Cardio‐ Metabochip | European | Sweden/ Norway/ Denmark |
| METISM | CAD/MI | METabolic Syndrome In Men | 224/1895 | 64.6(6.3)/ 58.7(7.3) | 0 | PMID:19223598 | Illumina Cardio‐ Metabochip | European | Kuopio/ Finland |
| MORGAM FIN | CAD | | | 1242/1242 | 64.5(7.3)/ 60.9(7.8) | 14.7 | PMID: 15561751 | Illumina Cardio‐ Metabochip | European | Finland |
| MORGAM FRA | CAD/MI | MONICA, Risk, | 183/183 | 57.6(3.0)/ 56.3(2.7) | 0 | PMID: 15561751 | Illumina Cardio‐ Metabochip | European | France |
| MORGAM GER | CAD/MI | Genetics, Archiving, | 215/215 | 64.5(7.8)/ 58.9(8.2) | 20.9 | PMID: 15561751 | Illumina Cardio‐ Metabochip | European | Germany |
| MORGAM ITA | CAD/MI | and Monograph | 151/151 | 61.3(9.3)/ 55.5(8.1) | 19.9 | PMID: 15561751 | Illumina Cardio‐ Metabochip | European | Italy |
| MORGAM UNK | CAD/MI | | | 164/164 | 59.7(4.1)/ 56.1(3.0) | 0 | PMID: 15561751 | Illumina Cardio‐ Metabochip | European | UK |
| PMB | CAD/MI | Pfizer-MGH-Broad | 922/4459 | 59.7(10.8)/ 57.6(10.3) | 45.7 | PMID: 23202125 | Illumina Cardio‐ Metabochip | European | Finnland/ Sweden |
| PopGen | CAD |  | 865/971 | 53.5(5.7)/ 54.7(15.2) | 30.5 | PMID: 18362232 | Illumina Cardio‐ Metabochip | European | Germany |
| SCARF SHEEP | CAD/MI |  | 1525/1892 | 57.6(7.3)/ 50.5(7.0) | 28 | PMID: 23202125 | Illumina Cardio‐ Metabochip | European | Sweden |
| STR | CAD | Swedish Twin Registry | 447/1272 | 78.9(9.7)/ 73.1(11.0) | 55.8 | PMID: 8981957 | Illumina Cardio‐ Metabochip | European | Sweden |
| ***addional 1000G*** |  |  |  |  |  |  |  |  |  |
| GerMIFS V | MI | German Myocardial Infarction Family Studies | 2459/1445 |  | 24.2/ 52.6 | - | Illumina HumanOmniExpress/Omniuni_2.5/Omni_Express1, FBRS, ZDHHC4 | European | Germany |
| GerMIFS VI | MI | German Myocardial Infarction Family Studies | 2496/1505 |  |  | - | llumina PsychChip_v1-1 | European | Germany |

Additional 26,508 CAD cases and 80,544 controls used for validation and extended meta-analysis. The sample size differs between SNPs for replication as not all SNPs are found in all studies.

# Supplement Table 2

DGIdb output for Coxib gene interactions

| **entrez_gene_symbol** | **gene_long_name** | **interaction_claim_source** | **interaction_types** | **drug_name** | **drug_primary_name** |
| --- | --- | --- | --- | --- | --- |
| ATP2A1 | ATPase, Ca++ transporting, cardiac muscle, fast twitch 1 | PharmGKB | n/a | PA448871 | CELECOXIB |
| ATP2A2 | ATPase, Ca++ transporting, cardiac muscle, slow twitch 2 | PharmGKB | n/a | PA448871 | CELECOXIB |
| ATP2A3 | ATPase, Ca++ transporting, ubiquitous | PharmGKB | n/a | PA448871 | CELECOXIB |
| BCAR1 | breast cancer anti-estrogen resistance 1 | PharmGKB | n/a | PA448871 | CELECOXIB |
| CA12 | carbonic anhydrase XII | GuideToPharmacologyInteractions | n/a | 2892 | CELECOXIB |
| CA12 | carbonic anhydrase XII | GuideToPharmacologyInteractions | n/a | 2894 | VALDECOXIB |
| CA12 | carbonic anhydrase XII | PharmGKB | n/a | PA448871 | CELECOXIB |
| CA9 | carbonic anhydrase IX | PharmGKB | n/a | PA448871 | CELECOXIB |
| CACNA1A | calcium channel, voltage-dependent, P/Q type, alpha 1A subunit | PharmGKB | n/a | PA448871 | CELECOXIB |
| CACNA1B | calcium channel, voltage-dependent, N type, alpha 1B subunit | PharmGKB | n/a | PA448871 | CELECOXIB |
| CACNA1C | calcium channel, voltage-dependent, L type, alpha 1C subunit | PharmGKB | n/a | PA448871 | CELECOXIB |
| CACNA1D | calcium channel, voltage-dependent, L type, alpha 1D subunit | PharmGKB | n/a | PA448871 | CELECOXIB |
| CACNA1E | calcium channel, voltage-dependent, R type, alpha 1E subunit | PharmGKB | n/a | PA448871 | CELECOXIB |
| CACNA1F | calcium channel, voltage-dependent, L type, alpha 1F subunit | PharmGKB | n/a | PA448871 | CELECOXIB |
| CACNA1G | calcium channel, voltage-dependent, T type, alpha 1G subunit | PharmGKB | n/a | PA448871 | CELECOXIB |
| CACNA1H | calcium channel, voltage-dependent, T type, alpha 1H subunit | PharmGKB | n/a | PA448871 | CELECOXIB |
| CACNA1I | calcium channel, voltage-dependent, T type, alpha 1I subunit | PharmGKB | n/a | PA448871 | CELECOXIB |
| CACNA1S | calcium channel, voltage-dependent, L type, alpha 1S subunit | PharmGKB | n/a | PA448871 | CELECOXIB |
| CACNA2D1 | calcium channel, voltage-dependent, alpha 2/delta subunit 1 | PharmGKB | n/a | PA448871 | CELECOXIB |
| CACNA2D2 | calcium channel, voltage-dependent, alpha 2/delta subunit 2 | PharmGKB | n/a | PA448871 | CELECOXIB |
| CACNA2D3 | calcium channel, voltage-dependent, alpha 2/delta subunit 3 | PharmGKB | n/a | PA448871 | CELECOXIB |
| CACNA2D4 | calcium channel, voltage-dependent, alpha 2/delta subunit 4 | PharmGKB | n/a | PA448871 | CELECOXIB |
| CACNB1 | calcium channel, voltage-dependent, beta 1 subunit | PharmGKB | n/a | PA448871 | CELECOXIB |
| CACNB2 | calcium channel, voltage-dependent, beta 2 subunit | PharmGKB | n/a | PA448871 | CELECOXIB |
| CACNB3 | calcium channel, voltage-dependent, beta 3 subunit | PharmGKB | n/a | PA448871 | CELECOXIB |
| CACNB4 | calcium channel, voltage-dependent, beta 4 subunit | PharmGKB | n/a | PA448871 | CELECOXIB |
| CASP3 | caspase 3, apoptosis-related cysteine peptidase | PharmGKB | n/a | PA448871 | CELECOXIB |
| CASP9 | caspase 9, apoptosis-related cysteine peptidase | PharmGKB | n/a | PA448871 | CELECOXIB |
| CDKN1A | cyclin-dependent kinase inhibitor 1A (p21, Cip1) | PharmGKB | n/a | PA448871 | CELECOXIB |
| CDKN1B | cyclin-dependent kinase inhibitor 1B (p27, Kip1) | PharmGKB | n/a | PA448871 | CELECOXIB |
| CTNNB1 | catenin (cadherin-associated protein), beta 1, 88kDa | PharmGKB | n/a | PA448871 | CELECOXIB |
| CYP2D6 | cytochrome P450, family 2, subfamily D, polypeptide 6 | PharmGKB | n/a | PA448871 | CELECOXIB |
| CYP3A4 | cytochrome P450, family 3, subfamily A, polypeptide 4 | PharmGKB | n/a | PA448871 | CELECOXIB |
| DDIT3 | DNA-damage-inducible transcript 3 | PharmGKB | n/a | PA448871 | CELECOXIB |
| ELN | elastin | DrugBank | other/unknown | DB00533 | ROFECOXIB |
| IGFBP3 | insulin-like growth factor binding protein 3 | PharmGKB | n/a | PA448871 | CELECOXIB |
| KCNQ1 | potassium voltage-gated channel, KQT-like subfamily, member 1 | PharmGKB | n/a | PA448871 | CELECOXIB |
| KCNQ2 | potassium voltage-gated channel, KQT-like subfamily, member 2 | PharmGKB | n/a | PA448871 | CELECOXIB |
| KCNQ3 | potassium voltage-gated channel, KQT-like subfamily, member 3 | PharmGKB | n/a | PA448871 | CELECOXIB |
| KCNQ4 | potassium voltage-gated channel, KQT-like subfamily, member 4 | PharmGKB | n/a | PA448871 | CELECOXIB |
| KCNQ5 | potassium voltage-gated channel, KQT-like subfamily, member 5 | PharmGKB | n/a | PA448871 | CELECOXIB |
| LTF | lactotransferrin | DrugBank | n/a | DB08439 | PARECOXIB |
| MMP9 | matrix metallopeptidase 9 (gelatinase B, 92kDa gelatinase, 92kDa type IV collagenase) | PharmGKB | n/a | PA448871 | CELECOXIB |
| PDK1 | pyruvate dehydrogenase kinase, isozyme 1 | PharmGKB | n/a | PA448871 | CELECOXIB |
| PDPK1 | 3-phosphoinositide dependent protein kinase-1 | DrugBank | inhibitor | DB00482 | CELECOXIB |
| PPARG | peroxisome proliferator-activated receptor gamma | PharmGKB | n/a | PA448871 | CELECOXIB |
| PTGS1 | prostaglandin-endoperoxide synthase 1 (prostaglandin G/H synthase and cyclooxygenase) | DrugBank | inhibitor | DB01283 | LUMIRACOXIB |
| PTGS1 | prostaglandin-endoperoxide synthase 1 (prostaglandin G/H synthase and cyclooxygenase) | PharmGKB | n/a | PA448871 | CELECOXIB |
| PTGS1 | prostaglandin-endoperoxide synthase 1 (prostaglandin G/H synthase and cyclooxygenase) | PharmGKB | n/a | PA451268 | ROFECOXIB |
| PTGS2 | prostaglandin-endoperoxide synthase 2 (prostaglandin G/H synthase and cyclooxygenase) | DrugBank | inhibitor | DB00482 | CELECOXIB |
| PTGS2 | prostaglandin-endoperoxide synthase 2 (prostaglandin G/H synthase and cyclooxygenase) | DrugBank | inhibitor | DB00533 | ROFECOXIB |
| PTGS2 | prostaglandin-endoperoxide synthase 2 (prostaglandin G/H synthase and cyclooxygenase) | DrugBank | inhibitor | DB00580 | VALDECOXIB |
| PTGS2 | prostaglandin-endoperoxide synthase 2 (prostaglandin G/H synthase and cyclooxygenase) | DrugBank | inhibitor | DB01283 | LUMIRACOXIB |
| PTGS2 | prostaglandin-endoperoxide synthase 2 (prostaglandin G/H synthase and cyclooxygenase) | DrugBank | inhibitor | DB01628 | ETORICOXIB |
| PTGS2 | prostaglandin-endoperoxide synthase 2 (prostaglandin G/H synthase and cyclooxygenase) | DrugBank | n/a | DB05095 | CIMICOXIB |
| PTGS2 | prostaglandin-endoperoxide synthase 2 (prostaglandin G/H synthase and cyclooxygenase) | GuideToPharmacologyInteractions | n/a | 2892 | CELECOXIB |
| PTGS2 | prostaglandin-endoperoxide synthase 2 (prostaglandin G/H synthase and cyclooxygenase) | GuideToPharmacologyInteractions | n/a | 2893 | ROFECOXIB |
| PTGS2 | prostaglandin-endoperoxide synthase 2 (prostaglandin G/H synthase and cyclooxygenase) | GuideToPharmacologyInteractions | n/a | 2894 | VALDECOXIB |
| PTGS2 | prostaglandin-endoperoxide synthase 2 (prostaglandin G/H synthase and cyclooxygenase) | GuideToPharmacologyInteractions | n/a | 2896 | ETORICOXIB |
| PTGS2 | prostaglandin-endoperoxide synthase 2 (prostaglandin G/H synthase and cyclooxygenase) | GuideToPharmacologyInteractions | n/a | 2897 | LUMIRACOXIB |
| PTGS2 | prostaglandin-endoperoxide synthase 2 (prostaglandin G/H synthase and cyclooxygenase) | MyCancerGenomeClinicalTrial | inhibitor | CELECOXIB | CELECOXIB |
| PTGS2 | prostaglandin-endoperoxide synthase 2 (prostaglandin G/H synthase and cyclooxygenase) | PharmGKB | n/a | PA10226 | VALDECOXIB |
| PTGS2 | prostaglandin-endoperoxide synthase 2 (prostaglandin G/H synthase and cyclooxygenase) | PharmGKB | n/a | PA164712669 | COXIBS |
| PTGS2 | prostaglandin-endoperoxide synthase 2 (prostaglandin G/H synthase and cyclooxygenase) | PharmGKB | n/a | PA164776853 | ETORICOXIB |
| PTGS2 | prostaglandin-endoperoxide synthase 2 (prostaglandin G/H synthase and cyclooxygenase) | PharmGKB | n/a | PA451268 | ROFECOXIB |
| PTGS2 | prostaglandin-endoperoxide synthase 2 (prostaglandin G/H synthase and cyclooxygenase) | TEND | n/a | ETORICOXIB | ETORICOXIB |
| PTGS2 | prostaglandin-endoperoxide synthase 2 (prostaglandin G/H synthase and cyclooxygenase) | TEND | n/a | LUMIRACOXIB | LUMIRACOXIB |
| PTGS2 | prostaglandin-endoperoxide synthase 2 (prostaglandin G/H synthase and cyclooxygenase) | TTD | inhibitor | DAP000737 | CELECOXIB |
| PTGS2 | prostaglandin-endoperoxide synthase 2 (prostaglandin G/H synthase and cyclooxygenase) | TTD | inhibitor | DAP000738 | ETORICOXIB |
| PTGS2 | prostaglandin-endoperoxide synthase 2 (prostaglandin G/H synthase and cyclooxygenase) | TTD | inhibitor | DAP000970 | LUMIRACOXIB |
| PTGS2 | prostaglandin-endoperoxide synthase 2 (prostaglandin G/H synthase and cyclooxygenase) | TTD | inhibitor | DAP001338 | ROFECOXIB |
| PTGS2 | prostaglandin-endoperoxide synthase 2 (prostaglandin G/H synthase and cyclooxygenase) | TTD | inhibitor | DAP001541 | VALDECOXIB |
| PTGS2 | prostaglandin-endoperoxide synthase 2 (prostaglandin G/H synthase and cyclooxygenase) | TdgClinicalTrial | n/a | APRICOXIB | APRICOXIB |
| PTGS2 | prostaglandin-endoperoxide synthase 2 (prostaglandin G/H synthase and cyclooxygenase) | TdgClinicalTrial | n/a | CELECOXIB | CELECOXIB |
| PTGS2 | prostaglandin-endoperoxide synthase 2 (prostaglandin G/H synthase and cyclooxygenase) | TdgClinicalTrial | n/a | CIMICOXIB | CIMICOXIB |
| PTGS2 | prostaglandin-endoperoxide synthase 2 (prostaglandin G/H synthase and cyclooxygenase) | TdgClinicalTrial | n/a | ETORICOXIB | ETORICOXIB |
| PTGS2 | prostaglandin-endoperoxide synthase 2 (prostaglandin G/H synthase and cyclooxygenase) | TdgClinicalTrial | n/a | LUMIRACOXIB | LUMIRACOXIB |
| PTGS2 | prostaglandin-endoperoxide synthase 2 (prostaglandin G/H synthase and cyclooxygenase) | TdgClinicalTrial | n/a | PARECOXIB | PARECOXIB |
| PTGS2 | prostaglandin-endoperoxide synthase 2 (prostaglandin G/H synthase and cyclooxygenase) | TdgClinicalTrial | n/a | ROFECOXIB | ROFECOXIB |
| VEGFA | vascular endothelial growth factor A | PharmGKB | n/a | PA448871 | CELECOXIB |

# Supplement Table 3

| **Phenotype** | **SNP** | **Lead SNP** | **LD lead** | **P-Value** | **PMID** |
| --- | --- | --- | --- | --- | --- |
| Waist Hip Ratio | rs6905288 | rs6905288 | 1 | 2.27x10^-26^ | 20935629 |
| HDL | rs6905288 | rs6905288 | 1 | 3.18x10^-4^ | 20686565 |
| Triglycerides | rs6905288 | rs6905288 | 1 | 3.54x10^-6^ | 20686565 |
| Systolic blood pressure | rs6905288 | rs6905288 | 1 | 4.51×10^-4^ | 21909115 |
| Diastolic blood pressure | rs6905288 | rs6905288 | 1 | 3.31×10^-4^ | 21909115 |
| Carotid intima media thickness | **rs4888383** | rs4888378 | 0.94 | 6.5x10^-7^ | 23152477 |
| Systolic blood pressure | **rs4888383** | rs4888378 | 0.94 | 1.8x10^-4^ | 21909115 |
| Pulse pressure | **rs4888383** | rs2865531 | 1 | 7.94x10^-6^ | 21909110 |
| Urinary albumin to creatinine ratio | **rs4888383** | rs4887829 | 0.82 | 2.80x10^-4^ | 21355061 |
| Microalbuminuria | **rs4888383** | rs10514393 | 0.92 | 6.00x10^-4^ | 21355061 |
| Blood metabolite concentrations | **rs556321** | rs633143 | 0.84 | 2.8x10^-4^ | 21886157 |
| Obesity | **rs556321** | rs633143 | 0.84 | 4.9x10^-4^ | 22484627 |

Pleiotropic effects related to SNPs. Association of CAD lead and LD SNPs with other traits. Bold indicates eQTL effects.

# Supplement Table 4

| **Phenotype** | **PMID** |
| --- | --- |
| Hypertension | 18516356, 17043442, 15710786 |
| HDL/Cholesterol/ Triglyceride | 20633016 |
| Weight gain | 17043442 |

Pleiotropic effects related to coxibs.

Supplement Table 5

| **RS ID** | **Studies missing** |
| --- | --- |
| rs7270354 | METSIM, MORGAM FIN, MORGAM FRA, MORGAM GER, MORGAM ITA, MORGAM UNK, POPGEN, SCARF, STR |
| rs4888383 | DILGOM, EPIC, FRISC, METSIM, MORGAM FIN, MORGAM FRA, MORGAM GER, MORGAM ITA, MORGAM UNK, PMB |
| rs6905288 | PMB |
| rs556321 |  |

Studies missing in meta-analysis per SNP.
